# Mortality and Causes of Death among Individuals in a National Opioid Agonist Treatment Cohort

**DOI:** 10.1159/ear/acrag005

**Published:** 2026-10-15

**Authors:** Tanja Schwarz, Judith Anzenberger, Martin Busch, Eva S. Schernhammer, Susanne Strohmaier

**Affiliations:** a Austrian National Public Health Institute (Gesundheit Österreich GmbH, GOEG), Vienna, Austria; b Doctoral Program Meduni Vienna, Medical University of Vienna, Vienna, Austria; c Department of Epidemiology, Center for Public Health, Medical University of Vienna, Vienna, Austria; d Department of Epidemiology, Harvard T.H. Chan School of Public Health, Boston, MA, USA

**Keywords:** Opioid-agonist treatment, Opioid use disorder, Mortality, Causes of death, Austria

## Abstract

**Introduction:**

People with opioid use disorder experience markedly elevated all-cause and cause-specific mortality. Opioid-agonist treatment (OAT) reduces mortality risk, and OAT populations across Europe are ageing, with cardiovascular, respiratory, and liver diseases accounting for a growing proportion of deaths. However, cause-specific mortality beyond overdose remains poorly characterized in large national cohorts.

**Methods:**

We conducted a retrospective cohort study including all 26,031 individuals (76% men) recorded in the Austrian national OAT registry between January 1, 2002, and December 31, 2019, linked to the national causes of death registry with follow-up through December 31, 2021. We calculated sex-specific age-standardized mortality rates per 100,000 person-years (2013 European Standard Population), standardized rate ratios (SRRs), and cause-specific standardized mortality ratios (SMRs) compared with the Austrian general population.

**Results:**

Across 278,959 person-years of follow-up, 2,358 deaths occurred. Cohort members had substantially higher mortality across all causes, with greater excess among women (SRR 7.06, 95% CI 6.45–7.72) than men (3.86; 3.69–4.04). Beyond substance use-related causes (SMR 33.2, 95% CI 31.4–35.2), the highest SMRs were found for viral hepatitis (63.8, 52.3–77.8), HIV (48.6, 39.0–60.6), and valvular heart diseases and endocarditis (28.4, 18.6–43.6). By the early to mid-40s, deaths from non-communicable diseases collectively exceeded those from substance use-related causes.

**Conclusion:**

Austria’s national OAT cohort experiences substantial excess mortality across all major causes, with high relative excess for substance use-related, infectious, and liver-related conditions. As OAT populations age, non-communicable diseases account for an increasing share of deaths. Future analyses linking the cohort to additional data sources will be needed to disentangle the underlying contributions to this excess mortality.

## Introduction

Evidence from systematic reviews and meta-analyses shows that people with opioid use disorder across Western industrialized countries face a substantially higher risk of death than the general population, from overdose, external causes, and somatic diseases linked to high-risk use and adverse social conditions [1, 2]. Opioid-agonist treatment (OAT) is the standard of care and typically combines prescribed long-acting opioids, such as methadone or buprenorphine [3], with additional clinical and psychosocial support. OAT is associated with lower all-cause and substance use-related mortality and with broader benefits at the individual, population, and societal levels, including improved infectious disease outcomes, mental and physical health, and reduced criminal activity [4–7]. At the same time, mortality and the distribution of specific causes of death among people in OAT are influenced by contextual factors such as treatment organization, medication profiles, and health system coverage.

Across Europe and other high-income regions, OAT populations are ageing, reflecting improved survival and demographic shifts [8–10]. This trend can be interpreted as a marker of treatment success, with fewer premature overdose deaths and more patients surviving into older age. As cohorts age, natural causes of death such as cardiovascular, respiratory, and liver disease, as well as cancer, account for a larger share of deaths, with pronounced differences in mortality between men and women [2, 4, 11, 12]. However, evidence from large European OAT cohorts remains limited, and analyses over extended follow-up periods are rare. Recent registry-based studies from Czechia, Denmark, and Norway have documented substantial variation in cause-specific mortality across settings, with leading causes of non-overdose death and mortality rates differing markedly between European countries [13, 14].

Understanding both the overall mortality burden and the cause-of-death profile in this population is crucial to inform targeted prevention, clinical management of comorbidities, and harm-reduction priorities, while generating country-specific evidence that can contextualize international findings and guide national policy. Austria provides a distinctive setting for such analyses as OAT is embedded in a universal, statutory health insurance system, with OAT medications reimbursed and care delivered mainly in outpatient settings by trained physicians, supporting a long-term treatment approach [9, 15]. We used a national Austrian OAT cohort from 2002 to 2021, linked to the Austrian national causes of death registry, to quantify and compare all-cause and cause-specific mortality with that of the general population and to examine how causes of death vary with age.

## Methods

### Setting and Participants

The study cohort was established linking national OAT prescription data and mortality records from Austria. The cohort includes all individuals who have ever received a prescription for OAT medications, as recorded in the national OAT registry [16]. For research purposes, a pseudonymized statistical sub-registry is used. The Austrian healthcare system provides universal coverage, and OAT has been fully reimbursed since 1989. Details on the prescribing system, data structure, and cohort construction are described in the Cohort Profile [17]. In short, treatment data, including longitudinal information on treatment episodes with initiation and discontinuation dates, were obtained from the statistical sub-registry and date and cause of death through the Austrian national causes of death registry. Linkage was conducted by authorized data custodians using the domain-specific personal health identifier (bPK-health). The analytical dataset includes all individuals who initiated OAT between 1 January 2002 and 31 December 2019, with mortality follow-up until 31 December 2021. Individuals contributed person time from cohort entry until death or end of follow-up, whichever occurred first. Ethical approval for this study was granted by the Ethics Committee of the Medical University of Vienna (reference No.: 1377/2022). There was no patient or public involvement in the study design.

### Cause of Death Categories

We categorized causes of death according to the International Classification of Diseases (ICD), 10th Revision (ICD-10). For 7% of deceased individuals in the OAT cohort, the cause of death could not be ascertained from the Austrian national causes of death registry. Causes of death were grouped into main categories that included mental and behavioural disorders due to psychoactive substance use (ICD-10 codes F11, F14, F15, F19), poisonings by drugs and medications (X40–X44, Y10–Y14), suicide (X60–X84 or Y87.0), other external causes of death (S00–T98), cancers (C00–D48, excl. C220 and C229), all liver-related diseases (B15–B19, K70–K77, C220, C229), cardiovascular diseases (I00–I99), HIV-related causes (B20–B24), and chronic respiratory disease (J40–J47).

We also defined a more detailed classification that followed the hierarchical structure of the ICD-10 following Santo et al. [4]. This classification comprised infectious and parasitic diseases (HIV, viral hepatitis); cancer (liver, pancreas, lung); endocrine, nutritional, and metabolic disorders (obesity and other hyperalimentation); mental and behavioural disorders (due to psychoactive substance use); circulatory system diseases (ischemic heart disease, valvular heart diseases and endocarditis, heart failure and cardiomyopathy); respiratory system diseases (chronic lower respiratory disease); digestive system diseases (liver diseases); and external causes (accidents, suicide, assault).

Drug-related deaths were classified according to two definitions: the broader categorization proposed by Santo et al. [4] and the narrower, harmonized European definition [18] proposed by the European Union Drugs Agency (EUDA; formerly EMCDDA). A detailed list of ICD-10 codes contributing to these definitions can be found in the online supplementary material (online suppl. Table S1; for all online suppl. material, see https://doi.org/10.1159/ear/acrag005).

### Statistical Analysis

We described person-years as well as main causes of death in the OAT cohort and the Austrian general population, overall and stratified by sex. Crude age-specific mortality rates and distributions of causes of death were visualized across age categories in both populations for the entire follow-up period as well as stratified into 5-year periods. Further, we calculated overall and sex-specific crude and age-standardized mortality rates (ASMRs) per 100,000 person-years for the main causes of death among individuals in the OAT cohort and the Austrian general population. To obtain the ASMRs, sex- and age-specific mortality rates in 5-year age groups in the OAT cohort and Austrian general population were weighted by the corresponding age distribution of the 2013 European Standard Population and summed across age strata [19] to allow for comparability across countries. Analyses were restricted to ages 15–69 years in both populations to enhance data stability as deaths and person-time were sparse in older and younger age groups in the OAT cohort. To compare cause-specific mortality between the OAT cohort and the general population, age-adjusted standardized rate ratios (SRRs) were calculated as the ratio of ASMRs between both populations. Corresponding 95% confidence intervals (CIs) were derived assuming a Poisson distribution for the observed number of deaths, using Byar’s approximation.

To examine more specific causes of death, we calculated crude rates but also used indirect standardization to calculate cause-specific standardized mortality ratios (SMRs) with 95% CIs. SMRs were chosen because case numbers for several detailed causes of death were low (<20), and indirect standardization provides more stable estimates under such conditions than direct rate standardization [20, 21]. The expected number of deaths was calculated by multiplying the person-years at risk in each stratum of the OAT cohort, defined by 5-year age group, sex, and calendar period, by the corresponding age-, sex-, and period-specific mortality rate in the Austrian general population for each calendar year between 2002 and 2021. The SMRs were then obtained as the ratio of observed to expected deaths, and 95% CIs were again calculated using Poisson (Byar) approximation.

Data preparation was conducted in SPSS (IBM Corp., Armonk, NY, USA), and statistical analyses were performed in *R*, version 4.4.1 (R Foundation for Statistical Computing, Vienna, Austria) using the packages dplyr, purrr, ggplot2, and scales. This study follows the Strengthening the Reporting of Observational Studies in Epidemiology (STROBE) guidelines for cohort studies; the completed checklist is provided in the online supplementary material.

## Results

The total study cohort included 26,031 individuals, of these 19,784 men (76%) and 6 247 women (24%), contributing a total of 278,959 person-years (PY) of follow-up. The median follow-up duration was 11.4 years (IQR 6.6–14.4), and the median age at cohort entry was 26 years (IQR 22–33). During follow-up, 2,358 individuals (9.1%) died, with causes of death identified for 2,201 (93%). Analyses were limited to individuals aged 15–69 years, among whom causes of death were available for 2,188 individuals who died during the 20-year observation period (see Table 1), corresponding to an overall crude mortality rate of 787 (95% CI 755–821) deaths per 100,000 PY (see online supplementary Tables S2 and S3). Drug-related deaths accounted for 803 out of 2,188 deaths with known cause (36.7%) using the EUDA definition and 1,172 deaths (53.6%) when applying the broader definition (Table 1, see online supplementary Table S1 for detailed definitions). Of the remaining deaths, overall the most common causes of deaths were liver disease (200 [9.1%]), suicide (181 [8.3%]), and other external causes of death (194 [8.9%]).

**Table 1.** Crude person-years and deaths in the OAT cohort and in the general population, by main causes of death^a^ (2002–2021)

​	Men		Women		Overall	
	OAT cohort	general population	OAT cohort	general population	OAT cohort	general population
Person-years	208,342	61,341,038	70,618	61,454,498	278,960	122,795,536
Deaths, *N*	1,868	242,358	476	128,089	2,344	370,447
Deaths (known cause)^b^, *N*	1,747 (100.0)	242,358 (100.0)	441 (100.0)	128,089 (100.0)	2,188 (100.0)	370,447 (100.0)
Mental and behavioural disorders due to psychoactive substance use, *n* (%)	588 (33.7)	2,464 (1.0)	144 (32.7)	744 (0.6)	732 (33.5)	3,208 (0.9)
Poisonings by drugs and medications, *n* (%)	293 (16.8)	1,202 (0.5)	73 (33.7)	535 (0.4)	366 (16.7)	1,737 (0.5)
Suicide, *n* (%)	152 (8.7)	13,963 (5.8)	29 (6.6)	4,147 (3.2)	181 (8.3)	18,110 (4.9)
Other external causes, *n* (%)	160 (9.2)	18,711 (7.7)	34 (7.7)	5,627 (4.4)	194 (8.9)	24,338 (6.6)
All liver-related, *n* (%)	148 (8.5)	20,942 (8.6)	52 (11.8)	6,586 (5.1)	200 (9.1)	27,528 (7.4)
Cancers, *n* (%)	105 (6.0)	79,969 (33.0)	36 (8.2)	62,538 (48.8)	141 (6.4)	142,507 (38.5)
Cardiovascular diseases, *n* (%)	97 (5.6)	55,453 (22.9)	16 (3.6)	21,468 (16.8)	113 (5.2)	76,921 (20.8)
HIV-related, *n* (%)	53 (3.0)	603 (0.2)	26 (5.9)	175 (0.1)	79 (3.6)	778 (0.2)
Chronic respiratory disease, *n* (%)	21 (1.2)	7,359 (3.0)	7 (1.6)	4,192 (3.3)	28 (1.3)	11,551 (3.1)
All other known causes, *n* (%)	130 (7.4)	41,692 (17.2)	24 (5.4)	22,077 (17.2)	154 (7.0)	63,769 (17.2)
Drug-related (EUDA), *n* (%)	637 (36.5)	2,627 (1.1)	166 (37.6)	795 (0.6)	803 (36.7)	3,422 (0.9)
Drug-related (broad), *n* (%)	945 (54.1)	11,365 (4.7)	227 (51.5)	4,508 (3.5)	1,172 (53.6)	15,873 (4.3)

EUDA, European Union Drugs Agency; HIV, human immunodeficiency virus; OAT, opioid-agonist treatment.

^a^ ICD-10: *mental and behavioural disorders due to psychoactive substance use* (excluding acute intoxication) = F11, F14, F15, F19 (96% in this category have F11.2, opioid dependence, or F19.2, multiple substance dependence); *poisonings by drugs and medications* (including acute intoxication, excluding intentional self-poisoning) = X40–X44, Y10–Y14, by specific substances (T40.0–T40.9, T42.3, T42.4, T42.6, T42.7, T43.6, T43.8, T43.9, T50.9); *suicide* = X60–X84 or Y87.0 (including intentional self-poisoning); *other external causes of death* = S00–T98 (all injuries, poisonings, and certain other consequences of external causes, excluding drug/medication intoxications and suicides); *cancers* = C00–D48, excluding C220 and C229; *all liver-related* = B15–B19 (viral hepatitis), K70–K77 (diseases of the liver), C220 (hepatocellular carcinoma), C229 (malignant neoplasm of liver, unspecified); *cardiovascular diseases* = I00–I99 (diseases of the circulatory system); *HIV-related* = B20–B24 (HIV disease); *chronic respiratory disease* = J40-J47 (chronic lower respiratory diseases); *drug-related (EUDA)* = see online supplementary Table S1; *drug-related (broad)* = see online supplementary Table S1.

^b^ A total of 156 deaths in the OAT cohort (15–69 years) had an unknown cause of death (121 men, 35 women).

We then examined age-specific mortality patterns in more detail (Fig. 1). In the OAT cohort, both age-specific rates and the distribution of causes showed that substance use-related causes dominated at younger ages, particularly mental and behavioural disorders due to psychoactive substance use, poisonings by drugs and medications, and other external causes. The frequent classification of deaths under mental and behavioural disorders due to psychoactive substance use is largely driven by World Health Organization rules for underlying cause-of-death coding, which prioritize substance dependence over immediate causes such as overdose or infection [22]. The contribution of substance use-related causes declined steadily with increasing age. From the mid-40s onward, liver-related mortality, cardiovascular diseases, and cancers increased sharply and made up an increasingly large share of overall deaths. By the early to mid-40s, deaths from non-communicable diseases collectively exceeded those from drug-related causes. In the general population, age-specific mortality and the proportion of cardiovascular and cancer deaths also increase from around 45 years but remain substantially lower than in the OAT cohort across all age groups. Additional visualizations stratified by 5-year follow-up periods highlight temporal shifts in cause-specific mortality patterns, although estimates for the first 10 years should be interpreted with caution due to low numbers of deaths in early follow-up periods (shown in online supplementary Fig. S1).

**Fig. 1.**
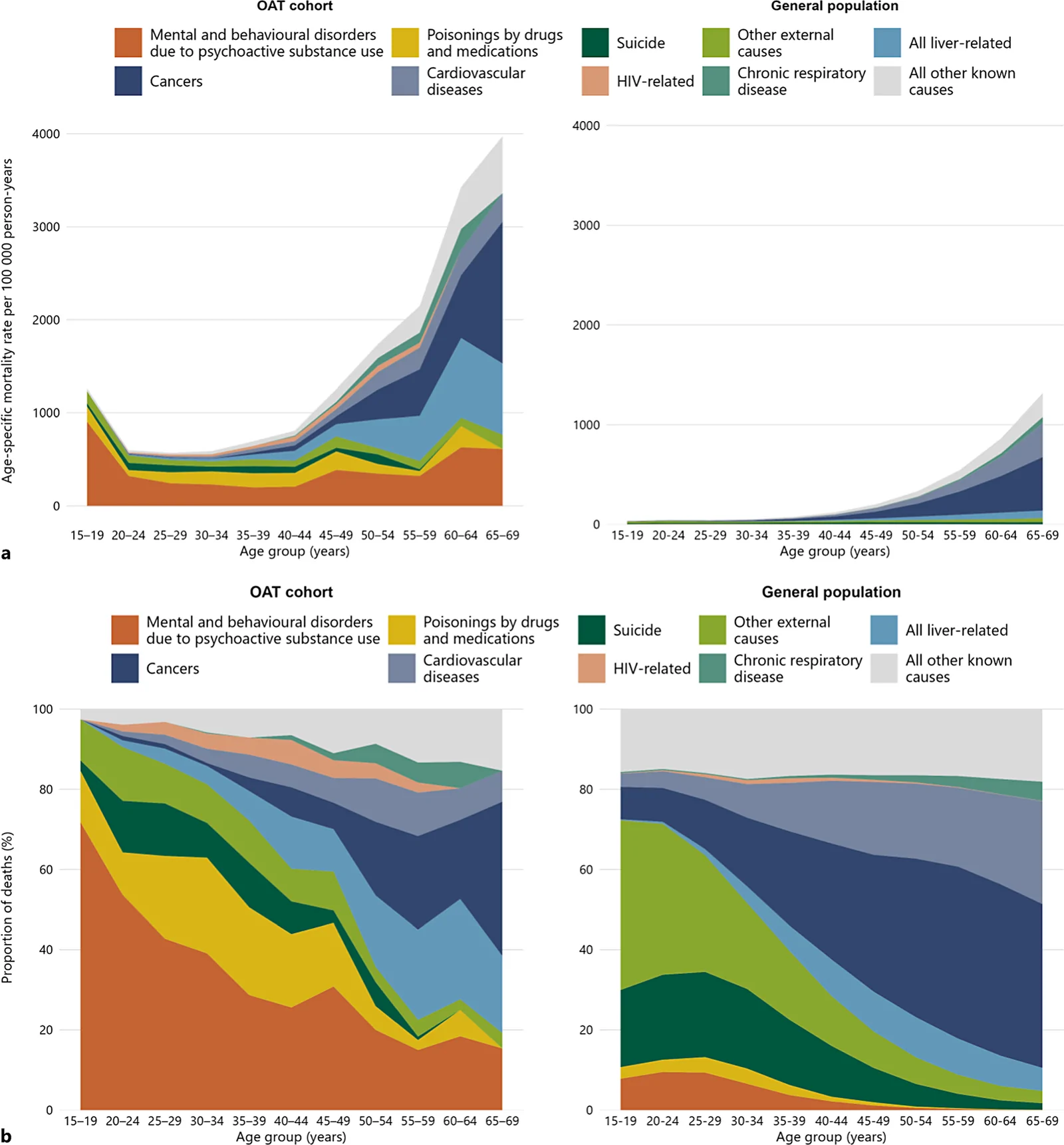
Age-specific mortality rates (**a**) and proportional mortality (**b**) among individuals in the OAT cohort and in the Austrian general population, by main causes of death (2002–2021). Cause of death categories are mutually exclusive. ICD-10: mental and behavioural disorders due to psychoactive substance use (excluding acute intoxication) = F11, F14, F15, F19 (96% in this category have F11.2, opioid dependence, or F19.2, multiple substance dependence); poisonings by drugs and medications (including acute intoxication, excluding intentional self-poisoning) = X40–X44, Y10–Y14 by specific substances (T40.0– T40.9, T42.3, T42.4, T42.6, T42.7, T43.6, T43.8, T43.9, T50.9); suicide = X60– X84 or Y87.0 (including intentional self-poisoning); other external causes of death = S00–T98 (all injuries, poisonings, and certain other consequences of external causes, excluding drug/medication intoxications and suicides); cancers = C00–D48, excluding C220 and C229; all liver-related = B15–B19 (viral hepatitis), K70–K77 (diseases of the liver), C220 (hepatocellular carcinoma), C229 (malignant neoplasm of liver, unspecified); cardiovascular diseases = I00–I99 (diseases of the circulatory system); HIV-related = B20–B24 (HIV disease); chronic respiratory disease = J40–J47 (chronic lower respiratory diseases).

### Age-Standardized Mortality Rates

Table 2 presents sex-specific ASMRs per 100,000 person-years and SRRs comparing individuals in the OAT cohort with the Austrian general population, standardized to the 2013 European Standard Population. Across all causes of death, mortality in the OAT cohort was substantially higher than in the general population (ASMR = 1 600 vs. 318 per 100,000 person-years; SRR = 5.03, 95% CI 4.83–5.24). Excess mortality was more pronounced among women (SRR = 7.06, 95% CI 6.45–7.72) than among men (SRR = 3.86, 95% CI 3.69–4.04) and particularly high for substance use-related causes. The SRR for mental and behavioural disorders due to psychoactive substance use was 93.45 (95% CI 85.41–102.25) in men and 359.68 (95% CI 300.9–429.94) in women. For poisonings by drugs and medications, the SRR was 69.52 (95% CI 61.18–78.98) in men and 93.38 (95% CI 73.12–119.24) in women. Mortality from all liver-related diseases was 7.28 (95% CI 6.19–8.56) times higher in men and 13.61 (95% CI 10.36–17.87) times higher in women compared with the general population. In contrast, excess mortality for cancers (SRR = 1.81, 95% CI 1.5–2.2 in men; 3.21, 95% CI 2.32–4.45 in women) and cardiovascular diseases (SRR = 1.22, 95% CI 1.00–1.49 in men; 1.47, 95% CI 0.9–2.4 in women) was less pronounced (Table 2).

**Table 2.** Overall and sex specific ASMRs per 100,000 person-years and SRRs with 95% CIs in the OAT cohort and in the general population, by main causes of death^a^ (2002–2021), standardized to ESP 2013

​	OAT cohort	General population	OAT cohort/general population
	ASMR	ASMR	SRR (95% CI)
Men			
Deaths (all)	**1,649.8**	**427.6**	**3.86 (3.69–4.04)**
Deaths (known cause)	**1,570.5**	**427.6**	**3.67 (3.50–3.85)**
Mental and behavioural disorders due to psychoactive substance use	372.7	4.0	93.45 (85.41–102.25)
Poisonings by drugs and medications	140.1	2.0	69.52 (61.18–78.98)
Suicide	58.1	23.0	2.52 (2.15–2.96)
Other external causes	100.2	31.2	3.21 (2.75–3.75)
All liver-related diseases	266.6	36.6	7.28 (6.19–8.56)
Cancers	259.6	143.2	1.81 (1.50–2.20)
Cardiovascular diseases	121.2	99.4	1.22 (1.00–1.49)
HIV-related	28.2	1.1	25.4 (19.18–33.64)
Chronic respiratory diseases	34.8	13.6	2.55 (1.66–3.91)
All other known causes	188.8	73.8	2.56 (2.15–3.04)
Drug-related (EUDA)	401.0	4.2	94.46 (86.62–103)
Drug-related (broad)	563.2	19.4	28.99 (27.13–30.98)
Women			
Deaths (all)	**1,512.5**	**214.3**	**7.06 (6.45–7.72)**
Deaths (known cause)	**1,371.7**	**214.3**	**6.40 (5.83–7.03)**
Mental and behavioural disorders due to psychoactive substance use	483.2	1.3	359.68 (300.9–429.94)
Poisonings by drugs and medications	98.2	1.1	93.38 (73.12–119.24)
Suicide	32.8	6.8	4.85 (3.37–6.99)
Other external causes	57.6	9.3	6.21 (4.43–8.70)
All liver-related diseases	149.9	11.0	13.61 (10.36–17.87)
Cancers	335.9	104.6	3.21 (2.32–4.45)
Cardiovascular diseases	53.3	36.2	1.47 (0.90–2.40)
HIV-related	31.9	0.6	57.73 (38.24–87.15)
Chronic respiratory diseases	65.8	7.3	9.02 (4.30–18.93)
All other known causes	62.9	37.0	1.7 (1.14–2.54)
Drug-related (EUDA)	516.0	1.4	361.83 (306.10–427.70)
Drug-related (broad)	601.7	7.5	80.54 (70.49–92.03)
Overall			
Deaths (all)	**1,600.2**	**318.0**	**5.03 (4.83–5.24)**
Deaths (known cause)	**1,510.2**	**318.0**	**4.75 (4.55–4.95)**
Mental and behavioural disorders due to psychoactive substance use	387.5	2.7	143.26 (132.21–155.23)
Poisonings by drugs and medications	126.0	1.6	80.16 (71.62–89.73)
Suicide	50.5	14.9	3.4 (2.94–3.93)
Other external causes	87.9	20.2	4.36 (3.78–5.02)
All liver-related diseases	243.6	23.5	10.37 (9.02–11.92)
Cancers	274.8	123.1	2.23 (1.89–2.63)
Cardiovascular diseases	107.6	66.8	1.61 (1.34–1.94)
HIV-related	29.5	0.9	33.16 (26.31–41.80)
Chronic respiratory diseases	41.1	10.4	3.97 (2.74–5.75)
All other known causes	161.7	54.9	2.95 (2.52–3.45)
Drug-related (EUDA)	416.5	2.9	145.12 (134.39–156.72)
Drug-related (broad)	557.1	13.3	41.76 (39.35–44.31)

ASMR, age-standardized mortality rate; CI, confidence interval; ESP 2013, European Standard Population (2013 version); EUDA, European Union Drugs Agency; HIV, human immunodeficiency virus; OAT, opioid-agonist treatment; SRR, standardized rate ratio.

^a^ ICD-10: *mental and behavioural disorders due to psychoactive substance use* (excluding acute intoxication) = F11, F14, F15, F19 (96% in this category have F11.2, opioid dependence, or F19.2, multiple substance dependence); *poisonings by drugs and medications* (including acute intoxication, excluding intentional self-poisoning) = X40–X44, Y10–Y14 by specific substances (T40.0–T40.9, T42.3, T42.4, T42.6, T42.7, T43.6, T43.8, T43.9, T50.9); *suicide* = X60–X84 or Y87.0 (including intentional self-poisoning); *other external causes of death* = S00–T98 (all injuries, poisonings, and certain other consequences of external causes, excluding drug/medication intoxications and suicides); *cancers* = C00–D48, excluding C220 and C229; *liver diseases* = B15–B19 (viral hepatitis), K70–K77 (diseases of the liver), C220 (hepatocellular carcinoma), C229 (malignant neoplasm of liver, unspecified); *cardiovascular diseases* = I00–I99 (diseases of the circulatory system); *HIV-related* = B20–B24 (HIV disease); *chronic respiratory disease* = J40-J47 (chronic lower respiratory diseases); *drug-related (EUDA)* = see online supplementary material; *drug-related (broad)* = see online supplementary material.

### Standardized Mortality Ratios

SMRs using the Austrian general population as the reference group are displayed in Figure 2. All-cause mortality among individuals in the OAT cohort was more than seven times higher than expected based on general population rates (SMR = 7.53, 95% CI 7.23–7.84). Excess mortality was higher in women (SMR = 12.36, 95% CI 11.30–13.53) than in men (SMR = 6.84, 95% CI 6.54–7.16).

**Fig. 2.**
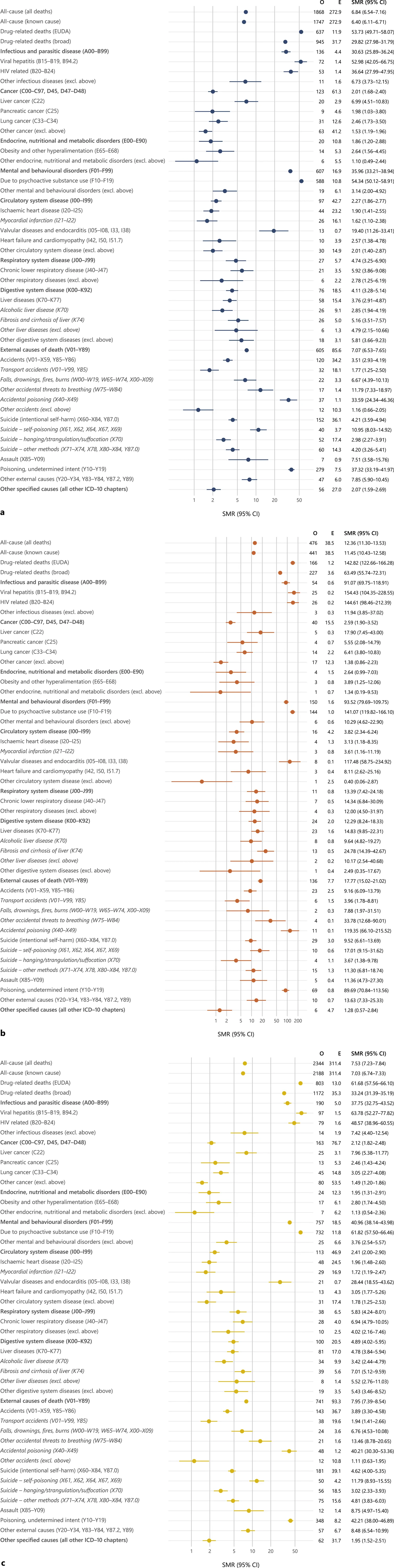
Observed (O) and expected (E) numbers of deaths and SMRs with 95% CIs, by detailed causes of death (2002–2021). **a** Men. **b** Women. **c** Overall.

The highest ratios were found for substance use-related causes and for mental and behavioural disorders due to psychoactive substance use (54.34, 95% CI 50.12–58.91 in men; 93.52, 95% CI 79.69–109.75 in women). Infectious diseases, particularly viral hepatitis (52.98, 95% CI 42.05–66.75 in men; 144.31, 95% CI 98.46–212.39 in women) and HIV-related causes (36.64, 95% CI 27.99–47.95 in men; 144.61, 95% CI 98.46–212.39 in women), also showed substantial excess mortality. High SMRs were further observed for liver diseases (3.76, 95% CI 2.91–4.87 in men; 14.8, 95% CI 9.85–22.31 in women) and for external causes, including suicide (4.21, 95% CI 3.59–4.94 in men; 9.52, 95% CI 6.61–13.69 in women) and accidental poisoning (33.59, 95% CI 24.34–46.36 in men; 119.35, 95% CI 66.10–215.52 in women). In contrast, relative differences were smaller for chronic somatic conditions such as cancers (2.01, 95% CI 1.68–2.40 in men, 2.59, 95% CI 1.90–3.52 in women) and circulatory system diseases (2.27, 95% CI 1.86–2.77 in men; 3.82, 95% CI 2.34–6.24 in women).

## Discussion

We examined mortality over a 20-year period among all individuals who entered OAT in Austria. Mortality across all examined causes was substantially higher than in the general population of the same age and sex, particularly in women. Substance use-related deaths remain the leading driver of excess mortality in this cohort. At the same time, the age-specific patterns indicate that with population ageing, non-communicable diseases are likely to account for an increasing share of excess mortality, and a considerable proportion of affected individuals now survive into ages in which chronic somatic conditions become more prominent.

These findings are broadly consistent with international evidence from other high-risk opioid using or treatment cohorts. The crude all-cause mortality rate in our study was 787 (95% CI 755–821) per 100,000 person years (online supplementary Tables S2 and S3), which is somewhat lower than the 964 (938–991) per 100,000 reported in an Australian OAT cohort [11], 1,340 (1,239–1,449) per 100,000 in a Croatian cohort study of opioid users in treatment [23], 1,375 (1,352–1,399) per 100,000 in a matched cohort of people who used illicit opioids in England [24], 1,400 (1,200–1,600) per 100,000 in a Norwegian OAT registry study [8], 1,820 (1,550–2,090) per 100,000 in a Norwegian take-home naloxone cohort [25], and a pooled rate of 1,100 (920–1,316) in OAT vs. 2,397 (1,992–2,883) out of OAT found in a systematic review and meta-analysis [4]. Although these crude comparisons place our cohort within the expected range for populations with a history of opioid use disorder, crude mortality rates remain difficult to compare across studies because of differences in age structure and background mortality. All the studies discussed above only report crude mortality rates and do not standardize to the European Standard Population 2013 (ESP2013). Standardization would enable more meaningful comparisons as SMRs are also influenced by underlying general population mortality. In our cohort, the all-cause SMR was 7.0 (6.7–7.3), indicating that individuals who entered OAT at some point experienced more than seven times the mortality expected on the basis of national age-specific and sex-specific mortality rates. Reported SMRs in comparable populations also show substantial variability. A pooled analysis across European cohorts reported an overall SMR of 10.0 (9.7–10.4), with marked variation between countries (3.5 in Malta to 21.5 in Poland) [26].

Gender differences in mortality were pronounced in our cohort, with women showing much higher relative excess mortality than men. Similar patterns have been reported in other OAT and opioid-using populations [11, 24, 27, 28]. This largely reflects the lower baseline mortality among women in the respective age range in the general population [29]. At the same time, it may also point to specific vulnerabilities among women with opioid use disorders, including gender-specific social and structural vulnerabilities [30, 31], exposure to violence [32], co-occurring mental health problems and psychiatric comorbidity [33–35], and sex-specific physiologic mechanisms [36, 37]. These disparities highlight the need for gender-sensitive approaches in OAT provision and in broader healthcare settings [35].

Beyond gender disparities, the mortality pattern in this population increasingly reflects the chronic disease burden seen in ageing opioid-using cohorts. Non-communicable diseases contributed substantially to mortality in this cohort, particularly liver-related conditions and cancers, reflecting the long-term consequences of hepatitis C infection, alcohol use, tobacco smoking, and advanced liver disease [28, 38]. Although highly effective direct-acting antivirals have been available in Europe for nearly a decade [39], barriers to HCV testing, referral, and treatment uptake persist among people who use substances [40, 41], leaving many with untreated or advanced disease [42]. Cancer-related mortality was also higher in the OAT cohort, in line with evidence from Australia [43], Norway [8, 28], and England [24], showing raised risks for lung, liver, and pancreas cancer in ageing opioid-using populations. These cancers are strongly linked to shared exposures such as smoking, blood-borne viruses, and alcohol use [44–46], as well as delayed healthcare seeking and low uptake of preventive care [47–49].

Importantly, the observed mortality patterns must be interpreted in the context of substantial changes in OAT provision and broader health care over the two decades covered by this study. Treatment guidelines [50–52], available OAT medications [7, 9, 53], access to harm reduction services [54–56], and the management of comorbid conditions such as HIV and hepatitis C [39, 57, 58] have evolved considerably over time. These changes are reflected in shifting mortality profiles across calendar periods and age groups, as detailed in the online supplementary analyses (online supplementary Fig. S1). While these developments have likely contributed to improved survival and an ageing OAT population, they also imply that an increasing proportion of individuals now live long enough to experience the long-term consequences of chronic substance use and accumulated somatic morbidity. However, these interpretations should be considered within the context of the descriptive analysis approach. While we document substantial excess mortality across all examined causes, we cannot explicitly disentangle the relative contribution of illicit opioid use, polysubstance use, or socioeconomic and behavioural factors to the observed excess mortality based on our available data.

### Strengths and Limitations

This national cohort study demonstrates the value of long-term, population-based follow-up for understanding mortality among people who enter OAT in Austria. By linking treatment and mortality registries with near-complete coverage over two decades, the analysis goes beyond a narrow focus on overdose deaths and shows that excess mortality increasingly reflects chronic and potentially preventable somatic conditions that extend beyond the immediate scope of addiction treatment. Furthermore, the utilization of the 2013 European Standard Population enables internationally comparable age-standardized mortality estimates.

The study has several limitations. The registry lacks information on behavioural and social factors such as injecting patterns, polysubstance use, mental health, or housing instability, which may influence mortality risk and hence would allow a more detailed characterization of the individuals included in the OAT cohort. Similarly, treatment trajectories in this population are often complex, involving multiple treatment episodes, interruptions, medication changes, and varying treatment durations. The present study intentionally focused on long-term cause-specific mortality at the cohort level to enable comparisons with international evidence based on comparable outcome measures. However, differences in drug markets, treatment systems, and harm-reduction coverage across countries might influence such direct comparisons and limit generalisability beyond the Austrian setting. Finally, missing information on causes of death among a small proportion of individuals in the OAT cohort likely leads to an underestimation of cause-specific SRRs and SMRs relative to the general population.

## Conclusions

Overall, the observed mortality pattern in this population reflects the chronic disease burden among people with opioid use disorders. Polysubstance use, ageing with long-term substance use, cumulative somatic burden [59], and high levels of socioeconomic disadvantage [30, 60] might interact to produce highly variable risk profiles within the OAT population. Disentangling these factors will require linking the OAT cohort to additional health and social data sources, potentially supported by emerging data infrastructures such as the European Health Data Space. Further, future detailed analyses of treatment episodes as time-varying exposures will allow to better understand how various treatment patterns contribute to the mortality risk in this population.

## Statement of Ethics

Ethical approval for this study was granted by the Ethics Committee of the Medical University of Vienna (reference No.: 1377/2022). There was no patient or public involvement in the study design. Data collection for this study is based on statutory registries maintained under the Austrian Narcotic Substances Act (Suchtmittelgesetz, SMG). Under Sections 8a(6) and 25(12) SMG, the rights set out in Articles 13, 14, 18, and 21 of the General Data Protection Regulation (including the right to be informed, the right of access, and the right to object) are explicitly excluded for data processed within these registries. Individual consent to register inclusion is therefore neither required nor applicable under Austrian law. This study uses data derived from these registries in accordance with Section 24dSMG, which permits their use for statistical and scientific analyses that do not produce person-identifiable results.

## Conflict of Interest Statement

The authors have no conflicts of interest to declare.

## Funding Sources

This research received no specific grant from any funding agency in the public, commercial or not-for-profit sectors. The Austrian National Public Health Institute (GOEG) is funded by the Federal Ministry of Labour, Social Affairs, Health, Care and Consumer Protection. The views expressed in this publication do not necessarily represent the position of the Austrian federal government. Collaborators from the Medical University of Vienna (Susanne Strohmaier) received support from the Austrian Academy of Science via Data:Research:Austria Programme (Project Identifier: DATA_2023_32_TopstatsMicrodata). The funders had no role in the design, data collection, data analysis, and reporting of this study.

## Author Contributions

T.S.: conceptualization, methodology, data curation, writing – original draft, writing – review and editing, and project administration. J.A.: resources and data curation. M.B.: writing – review and editing. E.S.S.: writing – review and editing and supervision. S.S.: conceptualization, methodology, writing – review and editing, and supervision.

## Data Availability

To protect privacy and confidentiality, the linkage of health data in Austria is provided under strict conditions governing its storage, retention, and utilization. Interested researchers are encouraged to contact the corresponding author to discuss potential collaborations and requirements. This includes obtaining approval from the Austrian Ministry of Health, which is the custodian of the data, for access to the dataset.
